# Fe(III) oxide respiration by a *Desulfovibrio* species lacking canonical outer-membrane electron transfer systems

**DOI:** 10.1128/mbio.02206-26

**Published:** 2026-10-09

**Authors:** Qin Cheng, Dawn E. Holmes, Dake Xu, Derek R. Lovley

**Affiliations:** 1 Key Laboratory for Anisotropy and Texture of Materials (Ministry of Education), Electrobiomaterials Institute, Northeastern University 12434, Shenyang, China; 2 State Key Laboratory of Digital Steel, Northeastern University 12434, Shenyang, China; 3 College of Medicine and Biological Information Engineering, Northeastern University 12434, Shenyang, China; 4 Department of Microbiology, University of Massachusetts 196202, Amherst, Massachusetts, USA; 5 Department of Physical and Biological Science, Western New England University 7473, Springfield, Massachusetts, USA; The University of Oklahoma, Norman, Oklahoma, USA

**Keywords:** *Desulfovibrio*, extracellular electron transfer, Fe(III) reduction, electron shuttle, riboflavin

## Abstract

*Desulfovibrio* species play an important role in the extracellular reduction of Fe(III) oxides and other metals in diverse environments, but their extracellular electron transfer (EET) mechanisms are poorly understood. We found that *Desulfovibrio* strain JY grew with H_2_ or lactate as the electron donor and poorly crystalline Fe(III) oxide as the electron acceptor, demonstrating the capacity to conserve energy from Fe(III) respiration despite the absence of genes for porin-cytochrome conduits, outer-surface cytochromes, or electrically conductive pili. Washed cell suspensions reduced Fe(III) citrate at rates comparable to that of *Shewanella oneidensis*, demonstrating the ability to effectively reduce soluble extracellular electron acceptors at the cell surface. Strain JY reduced the extracellular electron shuttles anthraquinone-2,6-disulfonate (AQDS) and riboflavin, which stimulated Fe(III) oxide reduction. Culture filtrates accelerated Fe(III) oxide reduction, and the reduction of Fe(III) oxide sequestered within porous alginate beads confirmed the presence of a shuttle. Riboflavin accumulated during growth on Fe(III) oxide, and electrochemical analysis and flavin measurements indicated that it was the primary shuttle. Genes encoding homologs of the *S. oneidensis* flavin exporter Bfe were more highly expressed during Fe(III) oxide respiration than during sulfate reduction. These results demonstrate that *Desulfovibrio* strain JY conserves energy to support growth from Fe(III) oxide reduction with a self-produced flavin electron shuttle, but without the canonical outer-membrane *c*-type cytochromes typically associated with EET in gram-negative bacteria. The findings expand the known diversity of microbial Fe(III) oxide respiration mechanisms and suggest that current genome-based prediction methods may substantially underestimate the diversity of electroactive microorganisms in natural environments.

**IMPORTANCE:**

Fe(III) oxide reduction is an important biogeochemical process influencing the cycling of carbon, nutrients, trace metals, and contaminants in anaerobic soils and sediments. The capacity for Fe(III) oxide reduction is commonly inferred from the presence of genes encoding porin-cytochrome conduits and outer-surface *c*-type cytochromes. The finding that a gram-negative microbe lacking these components can conserve energy to support growth from the reduction of Fe(III) oxides suggests that there is a much broader diversity of microbes capable of extracellular electron transfer than is generally considered. Genome-based strategies for elucidating the distribution and activity of electroactive microbes need revision to accommodate the expanding understanding of the range of mechanisms for electron transfer to insoluble extracellular electron acceptors.

## INTRODUCTION

Microbial Fe(III) oxide reduction exerts a major influence on the biogeochemistry of carbon, metals, and nutrients in diverse soils and sediments and can play a role in the bioremediation of organic and metal contaminants in the subsurface (1–3). Despite its importance, the mechanisms by which many microorganisms transfer electrons to insoluble Fe(III) oxides are poorly understood. A vast diversity of microorganisms have the capacity for extracellular electron transfer (EET), but detailed studies on EET mechanisms have been restricted to a few model microorganisms (3–6).

Mechanistic understanding of EET in gram-negative bacteria has largely been derived from studies of *Shewanella oneidensis* and *Geobacter sulfurreducens* (2, 7, 8). In both, electrons are transferred across the outer membrane via porin-cytochrome conduits composed of multi-heme *c*-type cytochromes embedded within membrane-spanning porin proteins. However, the downstream strategies differ. *S. oneidensis* primarily reduces Fe(III) oxides via flavin electron shuttles that are reduced at the cell surface by the porin-cytochrome conduits (7–10), whereas *G. sulfurreducens* relies on direct electron transfer mechanisms that require electrically conductive pili and outer-surface cytochromes (11–15).

The role of *Desulfovibrio* species in metal reduction is of interest because of their potential role in the reduction of Fe(III) oxides in sediments and remediation of uranium contamination via U(VI) reduction (16–18). Studies with *Desulfovibrio ferrophilus* demonstrated that genomic inferences of Fe(III) reduction mechanisms based on existing cytochrome-based models for EET can be misleading (19). It expresses two multi-heme *c*-type cytochromes homologous to the *G. sulfurreducens* nanowire-forming OmcS and also has genes for the proposed *G. sulfurreducens* OmcS nanowire assembly machinery (19). Yet, deletion of the OmcS genes, as well as the genes for the other five *c*-type cytochromes predicted to be associated with the outer membrane, had no impact on Fe(III) oxide reduction (19–21). Further analysis demonstrated that cells lacked outer-surface electrical contacts for the reduction of extracellular electron acceptors and relied on 2-amino-3-carboxy-1,4-naphthoquinone and related compounds functioning as internally reduced electron shuttles to transfer electrons to Fe(III) oxides (19). Notably, the genes for the *D. ferrophilus* outer-membrane cytochromes were more highly expressed in sulfate-grown cells rather than during Fe(III) oxide reduction (21), further suggesting a physiological role other than EET. These findings highlight that the presence of multi-heme, outer-surface cytochromes alone is not a reliable indicator of cytochrome-based EET, and that the EET mechanisms of gram-negative respiratory microbes can differ substantially from *Shewanella* and *Geobacter* species (19).

With the exception of *D. ferrophilus*, *Desulfovibrio* species previously evaluated (*D. vulgaris*, *D. baarsii*, *D. sulfodismutans*, and *D. desulfuricans*) cannot grow via Fe(III) oxide reduction (18). *Desulfovibrio* strain JY has been proposed to directly transfer electrons to other microbial species and electrodes despite an apparent lack of genes for porin-cytochrome conduits, other outer-surface *c*-type cytochromes, or electrically conductive pili (22). We hypothesized that strain JY was also capable of Fe(III) oxide reduction, but employed a non-canonical EET mechanism distinct from those in *S. oneidensis*, *G. sulfurreducens*, or *D. ferrophilus*.

The results presented here demonstrate that strain JY conserves energy to support growth from Fe(III) oxide respiration, employing a flavin electron shuttle for long-range electron transfer despite lacking canonical outer-membrane EET systems. These findings expand the known diversity of Fe(III) respiration mechanisms in gram-negative bacteria and further demonstrate that current strategies for genome-based predictions do not accurately capture the diversity of microorganisms capable of extracellular electron transfer.

## RESULTS AND DISCUSSION

### Strain JY growth via Fe(III) oxide reduction

**Fig 1:**
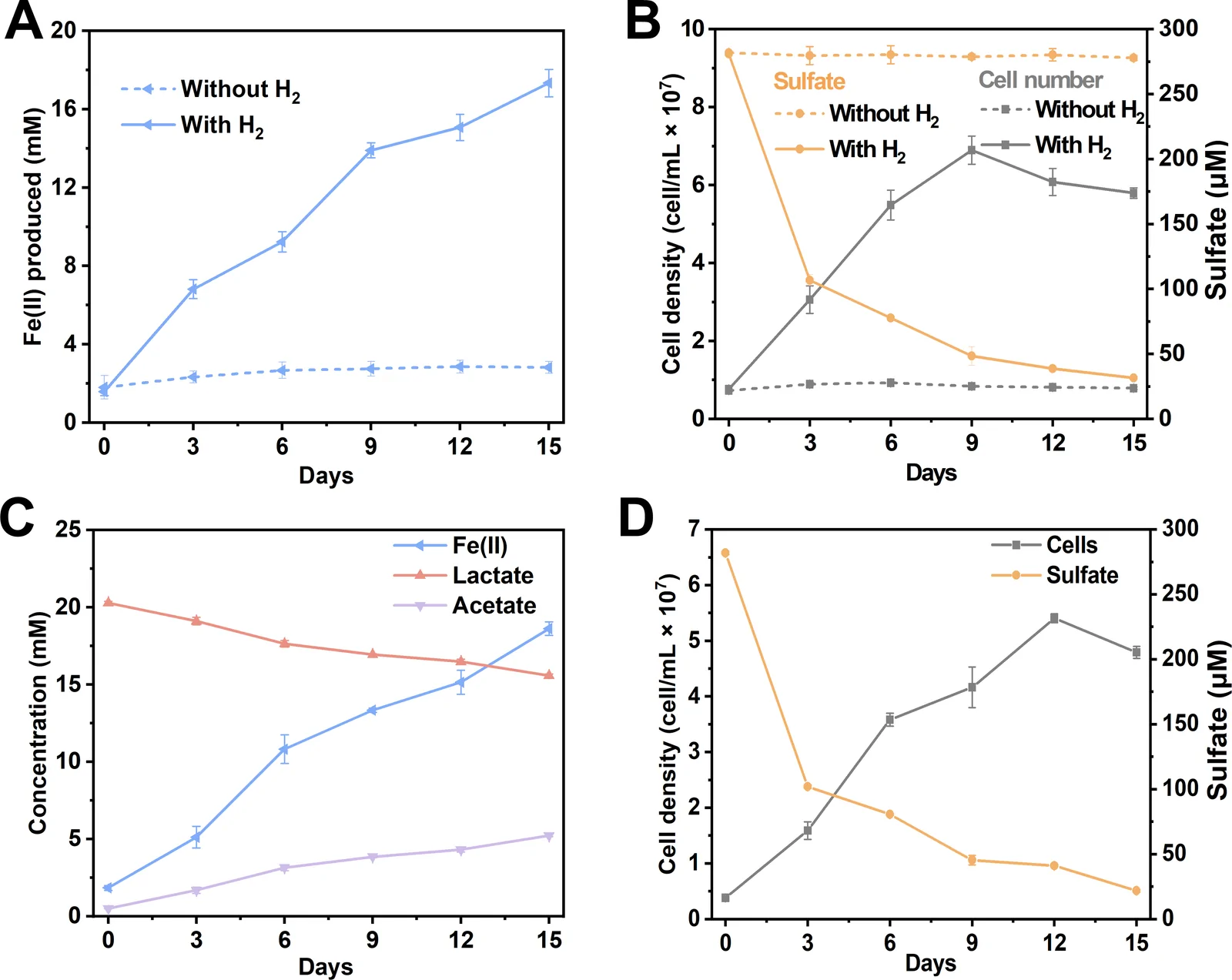
Strain JY growth and Fe(III) and sulfate reduction with H_2_ or lactate as the electron donor. (**A**) H_2_ utilization with the reduction of Fe(III) to Fe(II) in medium with poorly crystalline Fe(III) oxide as the primary electron acceptor. Sulfate introduced with the mineral salt solution was present at low concentrations. Acetate, which cannot be used as an electron donor, was added as a carbon source. (**B**) Cell numbers and sulfate concentrations over time in the incubation in panel** A**. (**C**) Lactate metabolism to acetate with the reduction of Fe(III) to Fe(II) in medium with poorly crystalline Fe(III) oxide as the primary electron acceptor. (**D**) Cell numbers and sulfate concentrations over time in the incubation in panel **C**. The results are the mean and standard deviation for triplicate incubations of each treatment.

Strain JY grew with Fe(III) oxide as the electron acceptor (Fig. 1). H_2_-dependent growth (Fig. 1A and B) demonstrated that energy could be conserved from Fe(III) respiration. Lactate was incompletely oxidized to acetate and carbon dioxide with the reduction of Fe(III) (Fig. 1C and D).

Previous studies demonstrated that growth of *D. desulfuricans* in Fe(III) oxide medium was coincident with the reduction of the ca. 300 µM sulfate introduced into the medium with the trace metal mix (18). Fe(III) reduction continued after the sulfate was reduced, but there was no further growth. In contrast, strain JY continued to grow after reduction of the trace sulfate stopped (Fig. 1D). Subsequent transfers in medium without Fe(III) oxide demonstrated that the sulfate available in the medium could not support sustained growth or lactate metabolism (Fig. S1).

These results demonstrated that strain JY conserves energy to support growth from Fe(III) oxide reduction. This contrasts with the previous finding that, with the exception of *D. ferrophilus* (19, 21), most *Desulfovibrio* species that have been evaluated (*D. vulgaris*, *D. baarsii*, *D. sulfodismutans*, and *D. desulfuricans*) (18) do not grow with Fe(III) oxide as the electron acceptor.

### Reduction of extracellular electron acceptors at the outer cell surface

**Fig 2:**
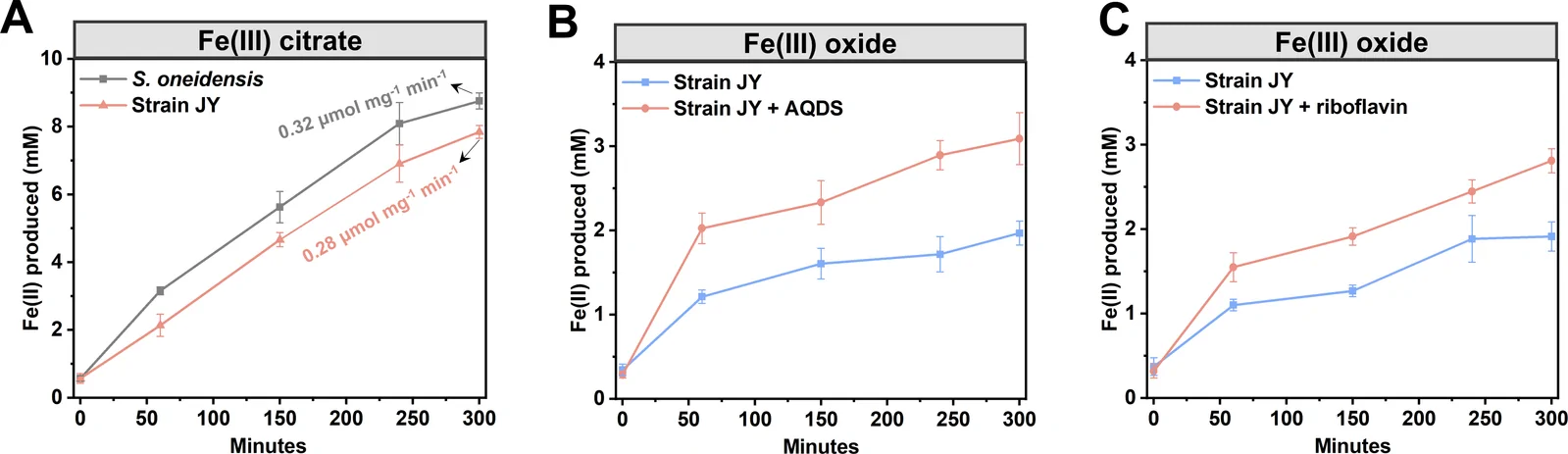
Ability of strain JY washed cell suspensions to reduce extracellular electron acceptors. (**A**) Fe(II) production from Fe(III) citrate reduction by strain JY and the positive control of *S. oneidensis*. (**B**) AQDS stimulation of strain JY Fe(III) oxide reduction. (**C**) Riboflavin stimulation of strain JY Fe(III) oxide reduction. The results are the mean and standard deviation for triplicate incubations of each treatment.

Washed cell suspensions of strain JY reduced Fe(III) citrate at a rate that was ca. 90% of washed cell suspensions of *S. oneidensis* (Fig. 2A), demonstrating a substantial capacity for reduction of extracellular electron acceptors at the outer cell surface. This result contrasts with *D. ferrophilus*, which relies on intracellularly reduced electron shuttles for EET. It cannot reduce Fe(III) citrate, which due to its size and charge, does not readily cross the outer membrane of gram-negative cells (19).

Further evidence for the ability of strain JY to reduce electron acceptors at the outer cell surface was the finding that amendments of anthraquinone-2,6-disulfonate (AQDS) stimulated reduction of Fe(III) oxides by washed cell suspensions of strain JY (Fig. 2B). AQDS must be reduced at the outer cell surface because of its size and polarity. For microbes capable of transferring electrons to the outer-cell surface, AQDS is a very effective electron shuttle for Fe(III) oxide reduction (3, 23).

Flavins function as extracellularly reduced electron shuttles for Fe(III) oxide reduction in a similar manner (2, 7, 8). Like AQDS, riboflavin stimulated Fe(III) oxide reduction in washed cell suspensions of strain JY (Fig. 2C).

These results demonstrate that strain JY has the ability to reduce a diversity of soluble redox-active molecules at the outer cell surface despite lacking the canonical porin-cytochrome conduits that are important in electron transfer across the outer membrane in intensively studied electroactive gram-negatives like *Shewanella* and *Geobacter* species.

### Evidence for riboflavin as a physiological electron shuttle

The riboflavin stimulation of Fe(III) oxide reduction suggested that flavins might function as self-produced electron shuttles during growth on Fe(III) oxides. Several independent observations based on studies like those that led to the identification of self-secreted flavins as electron shuttles for *Shewanella* species (24–26) supported this possibility.

**Fig 3:**
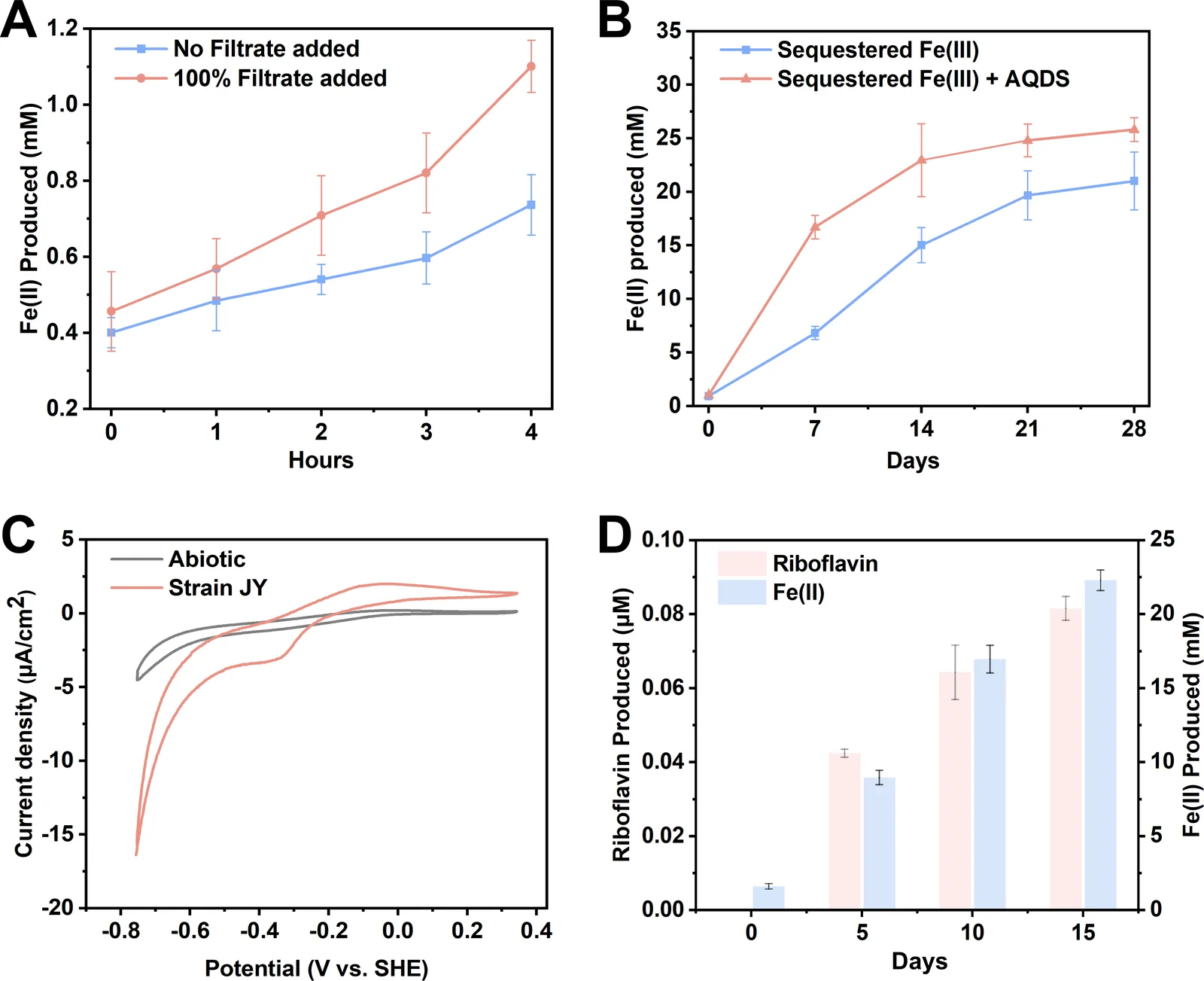
Evidence for the involvement of excreted riboflavin as an electron shuttle for strain JY Fe(III) oxide reduction. (**A**) Stimulation of washed cell suspension Fe(III) oxide reduction with culture filtrate from Fe(III) oxide-grown cells. (**B**) Fe(II) produced from strain JY reduction of poorly crystalline Fe(III) oxide sequestered in porous alginate beads in the presence or absence of 50 μM AQDS. (**C**) Cyclic voltammograms (scan rate 10 mV/s versus standard hydrogen electrode [SHE]) of culture filtrates from strain JY grown for 15 days on Fe(III) oxide. (**D**) Concentration of extracellular riboflavin and production of Fe(II) in Fe(III) oxide cultures of strain JY. The results in panels A, B, and D are the mean and standard deviation for triplicate incubations of each treatment.

Fe(III) oxide reduction was stimulated when washed cells were resuspended in cell-free filtrates collected from Fe(III) oxide-grown cultures (Fig. 3A), as would be expected if a self-produced electron shuttle accumulated during growth on Fe(III) oxide. Strain JY also reduced Fe(III) oxide sequestered in porous alginate beads (Fig. 3B), demonstrating the capacity to reduce Fe(III) oxide without the need for direct cell contact. Although AQDS accelerated the initial rate that Fe(III) oxides in the beads were reduced, the rate of unamended incubations increased over time, consistent with the accumulation of a self-produced extracellular electron shuttle.

Cyclic voltammetry of culture filtrates from Fe(III) oxide-grown cells revealed oxidation and reduction peaks at approximately −200 mV versus SHE (Fig. 3C), similar to the reported midpoint potential of riboflavin (27). Flavin analysis demonstrated a progressive accumulation of riboflavin during growth on Fe(III) oxide (Fig. 3D). Flavin mononucleotide, which is the most abundant flavin found in *S. oneidensis* cultures (9, 25), was not detected.

The strain JY genome contains two homologs (K9F62_11825 and K9F62_04930; Fig. S2) of the gene for Bfe, which *S. oneidensis* requires for extracellular flavin secretion (9). Transcriptomic analysis revealed that K9F62_11825 transcripts were 4.1-fold higher (*P* = 5.4 × 10^−14^) in cells grown on Fe(III) oxide versus cells grown with sulfate as the electron acceptor (Table S1). The K9F62_04930 transcripts were 4.2-fold (*P* = 7.7 × 10^−19^) higher in Fe(III) oxide-grown cells (Table S1). The gene (K9F62_07545) for RibBA, a 3,4-dihydroxy-2-butanone-4-phosphate synthase/GTP cyclohydrolase II that catalyzes the first two committed steps of riboflavin biosynthesis, was more highly expressed (2.2-fold; *P* = 1.9 × 10^−13^) in Fe(III) oxide-grown cells than cells grown with sulfate (Table S1). These transcriptome results further support the concept that exported riboflavin is an important electron shuttle during Fe(III) oxide reduction.

The presence of flavin exporter genes, their higher expression during growth on Fe(III) oxide, the accumulation of extracellular riboflavin over time in Fe(III) oxide-grown cells, the reduction of Fe(III) oxide occluded from direct cell contact, the stimulation of Fe(III) oxide reduction by Fe(III) oxide-grown culture filtrates or added riboflavin, and the ability to reduce soluble electron acceptors at the outer cell surface all indicate that, like *S. oneidensis*, strain JY employs a self-produced flavin to enhance Fe(III) oxide reduction. The distinction is that strain JY lacks the porin-cytochrome conduits of *S. oneidensis* to transfer electrons to the outer cell surface.

### Further transcriptional analysis of Fe(III) oxide reduction

Due to the lack of recognizable trans-outer-membrane electron transfer systems in the strain JY genome, transcriptomes from Fe(III) oxide- and sulfate-grown cells were compared to identify possible alternative pathways. None of the genes more highly expressed during growth on Fe(III) oxide could confidently be assigned a role in electron transfer across the outer membrane (Table S1). Further functional studies, including targeted gene deletions and biochemical characterization of outer-membrane proteins, to search for possible outer-membrane electron transfer candidates are required. However, genetic manipulation of strain JY is currently limited by the absence of protocols for DNA delivery, selectable-marker expression, and targeted genome modification.

### Implications

The results demonstrate that *Desulfovibrio* strain JY employs a previously unrecognized EET strategy for a gram-negative respiratory bacterium, in which the reduction of extracellular electron acceptors at the cell surface is achieved without outer-surface c-type cytochromes or outer membrane porin-cytochrome conduits. Multiple lines of evidence also indicate that strain JY secretes riboflavin to extend this outer-surface EET capacity to reduce Fe(III) oxide at a distance from the cell. The strain JY Fe(III) oxide reduction mechanism is distinct from that of *Geobacter* species, which require direct contact, distinct from *S. oneidensis*, which employs outer-surface *c*-type cytochromes to reduce its flavin electron shuttles, and distinct from *D. ferrophilus*, which relies on an internally reduced electron shuttle (2, 3, 7, 8, 19).

Models for microbial Fe(III) reduction in soils and sediments have largely been extrapolated from the cytochrome-based mechanisms of *Shewanella* and *Geobacter* (2, 3, 8). The findings presented here, together with recent studies on *D. ferrophilus* (19), demonstrate that such models are incomplete and that the presence or absence of outer-surface *c*-type cytochromes is not a reliable predictor of EET strategy. These results highlight the need for additional functional analyses to identify the molecular basis of EET in diverse microorganisms to enable development of an improved framework for interpreting genomic data that can capture the full diversity of microbes capable of EET.

## MATERIALS AND METHODS

### Microbial culturing

Strain JY was kindly provided by Shiling Zheng (Key Laboratory of Coastal Biology and Biological Resources Utilization, Yantai Institute of Coastal Zone Research, Chinese Academy of Sciences) (22). Cultures were routinely maintained anaerobically as previously described (22) at 30°C in 10 mL of NB medium (28) dispensed into 28 mL anaerobic pressure tubes and incubated under an N_2_/CO_2_ (80:20, vol/vol) atmosphere. DL-lactate (40 mM) served as the electron donor and sulfate (20 mM) as the electron acceptor. NB medium contained the following components (per liter of deionized water): KH_2_PO_4_, 0.42 g; K_2_HPO_4_, 0.22 g; NH_4_Cl, 0.2 g; KCl, 0.38 g; NaCl, 0.36 g; CaCl_2_·2H_2_O, 0.04 g; MgSO_4_·7H_2_O, 0.1 g; NaHCO_3_, 1.8 g; Na_2_CO_3_, 0.5 g; 1.0 mL of 1 mM Na_2_SeO_4_, 15.0 mL vitamin solution (29), and 10.0 mL of NB trace mineral solution (29). The tubes were sealed with thick butyl rubber stoppers.

### Fe(III) reduction

To evaluate the potential for growth using Fe(III) oxide as the electron acceptor, poorly crystalline Fe(III) oxide, prepared as previously described (30), was added to pressure tubes at a final concentration of 100 mmol/L. H_2_ or DL-lactate (20 mM) was provided as the electron donor. Acetate (10 mM), which strain JY cannot use as an electron donor (31), was added as a carbon source. To minimize sulfate carryover, the inoculum was pre-grown in medium containing only 5 mM sulfate.

### Cell suspension

To assess the ability of strain JY cell suspensions to reduce Fe(III) citrate or Fe(III) oxide, cells were pre-grown in NB medium with DL-lactate (40 mM) as the electron donor and sulfate (20 mM) as the electron acceptor. Cells were harvested by centrifugation (7,520 × *g*, 10 min, 4℃), resuspended in anaerobic bicarbonate buffer (NaHCO_3_, 2.5 g/L; NaH_2_PO_4_·H_2_O, 0.6 g/L; NH_4_Cl, 0.25 g/L; KCl, 0.1 g/L; NaCl, 1.75 g/L), centrifuged again, and finally resuspended in approximately 3 mL of anaerobic minimal bicarbonate buffer (NaHCO_3_, 2.5 g/L; NH_4_Cl, 0.25 g/L; NaH_2_PO_4_·H_2_O, 0.6 g/L). Cell protein concentrations were determined using the Bradford assay (32). Aliquots of the cell suspension (0.6–0.8 mL) were added to 10 mL of anaerobic medium to provide approximately 0.80 mg cell protein per sample, and either Fe(III) citrate (10 mM) or Fe(III) oxide (10 mmol/L) was provided as the electron acceptor and DL-lactate (2.5 mM) as the electron donor. *Shewanella oneidensis* MR-1 served as positive control. Cells of *S. oneidensis* MR-1 were pre-grown in freshwater medium, with DL-lactate (25 mM) as the electron donor and fumarate (40 mM) as the electron acceptor, and processed in the same manner as strain JY. When indicated, incubations were supplemented with AQDS (50 μM) or riboflavin (10 μM). Cell suspensions were incubated at 30℃ and sampled at 0, 60, 150, 240, and 300 min for Fe(II) analysis.

### Assay of electron shuttles involved in Fe(III) reduction

To determine whether strain JY produced soluble compounds capable of stimulating Fe(III) oxide reduction, the effect of culture filtrates on Fe(III) oxide reduction was evaluated. Cultures grown on lactate-Fe(III) oxide medium were filtered through a 0.22-μm polyvinylidene difluoride membrane. The filtrate was exposed to air with stirring for 1 h to oxidize dissolved Fe(II), after which the resulting Fe(III) precipitates were removed by filtration. The filtrate was then mixed with fresh medium at various dilution ratios. Aliquots (10 mL) of the filtrate-medium mixtures were transferred to pressure tubes, sparged with N_2_/CO_2_ (80:20) to remove dissolved oxygen, and sealed with butyl rubber stoppers. Poorly crystalline Fe(III) oxide (10 mmol/L) was added from a concentrated anaerobic stock. A washed cell suspension of strain JY pre-grown in lactate-sulfate medium was then inoculated into the tubes. Samples were collected hourly for Fe(II) analysis.

To investigate whether strain JY secretes soluble electron shuttles during extracellular electron transfer, synthetic poorly crystalline Fe(III) oxide (ca. 100 mmol/L) was incorporated into porous alginate beads as previously described (33). Briefly, Fe(III) oxide was stirred with a solution of sodium alginate and sodium *meta-*periodate, and the mixture was dropped into a CaCl_2_ solution (100 mM) containing polyethyleneimine (0.5% [wt/vol], number average molecular weight 15,000) to form 5 mm diameter beads with Fe(III) oxide entrapped within the alginate matrix. The final concentration of entrapped Fe(III) oxide was approximately 100 mmol/L. These Fe(III)-oxide containing beads were added to anaerobic cultures of strain JY. In selected incubations, AQDS (50 µM) was added at the same time as the entrapped Fe(III) oxide beads. Fe(II) production was monitored over time to assess reduction of the entrapped Fe(III) oxide.

Cyclic voltammetry was performed to characterize the redox properties of soluble electron shuttles produced by strain JY during growth with Fe(III) oxide as the electron acceptor. Electrochemical measurements were conducted using an electrochemical workstation (Reference 600+, Gamry Instruments, USA). A three-electrode configuration was employed, consisting of a tin-doped In_2_O_3_ electrode (surface area: 2 cm^2^) as the working electrode, a saturated calomel electrode as the reference electrode, and a platinum plate electrode (10 mm × 10 mm × 1 mm) as the counter electrode. Prior to use, the working electrode was cleaned by sequential sonication for 10 min each in acetone, anhydrous ethanol, and ultrapure water, and then dried under a stream of N_2_. Culture filtrates were sparged with N_2_/CO_2_ (80:20) to maintain anaerobic conditions. To eliminate interference from riboflavin present in the vitamin solution, strain JY was grown in medium lacking vitamin supplement prior to collection of culture filtrates for cyclic voltammetry analysis.

### RNA sequencing

Triplicate cultures of strain JY grown with lactate as the electron donor and sulfate or Fe(III) oxide as the sole electron acceptor were harvested during the mid-logarithmic phase for transcriptomic analysis. Cells were collected by centrifugation in 50 mL conical tubes at 5,000 × *g* for 15 min at 4℃. After centrifugation, cell pellets were flash-frozen in liquid nitrogen and stored at −80℃ until RNA extraction.

RNA was extracted from pellets collected from lactate-Fe(III) oxide grown cells using a previously described method (34). Total RNA from lactate-sulfate grown cells was extracted using TRIzol reagent (Invitrogen, Carlsbad, CA, USA) according to the manufacturer’s instructions. RNA quantity, purity, and integrity were assessed using a NanoDrop 2000 spectrophotometer (Thermo Fisher Scientific, USA) and a Bioanalyzer 2100 system (Agilent Technologies, USA).

Ribosomal RNA was removed from RNA samples using the Zymo-Seq RiboFree Total RNA Library Kit (Zymo Research, USA). Strand-specific cDNA libraries were constructed following the manufacturer’s protocol, including first-strand synthesis with SuperScript III and second-strand synthesis using DNA polymerase I and RNase H (New England Biolabs, USA) with dUTP incorporation. Libraries were purified using AMPure XP beads (Beckman Coulter, USA), quantified using a Bioanalyzer 2100, and sequenced on an Illumina NovaSeq 6000 platform (Illumina, USA) by Shanghai Personal Biotechnology Co., Ltd. (Shanghai, China).

### Bioinformatics analysis

The predicted proteome of strain JY was searched for proteins containing the canonical CXXCH heme-binding motif of *c*-type cytochromes. The subcellular localization of the identified candidates was subsequently predicted using PSORTb (35). The genome of strain JY was screened for homologs of the *bfe* gene from *S. oneidensis* MR-1, which encodes a membrane transporter involved in flavin export (9). The Bfe protein sequence from *S. oneidensis* MR-1 (UniProt accession: Q8EIX5) was used as the query sequence. BLASTp searches were performed using BLAST+ with default parameters and the BLOSUM62 substitution matrix. Hits with an e-value ≤1e−5 and alignment coverage ≥70% were considered potential homologs. Putative Bfe homologs were further evaluated by reciprocal BLASTp searches against the NCBI nonredundant (nr) database to determine whether Bfe proteins were recovered as the top matches. Multiple-sequence alignments of the identified Bfe homologs were performed with ClustalW (v2.1) (36) and visualized using ESPript (v3.0) (37).

Raw sequencing reads in FASTQ format were quality filtered using fastp (v0.22.0) (38). Adapter sequences, reads containing poly-N regions, and low-quality reads were removed to generate high-quality clean reads for downstream analyses. Clean reads were mapped to the strain JY genome with Bowtie2 (39), and gene-level read counts were determined using HTSeq (v0.9.1) (40). Gene expression levels were normalized as fragments per kilobase of transcript per million mapped reads (FPKM) to facilitate comparisons among genes and samples. Differentially expressed genes (DEGs) between growth conditions were identified using DESeq2 (v1.38.3) (41). Genes with an absolute log_2_ fold change >1 and a Benjamini–Hochberg adjusted *P*-value <0.05 were considered differentially expressed.

### Analytical techniques

Sulfate concentrations were determined by ion chromatography using the Dionex AS-DV System (Thermo Fisher Scientific, USA). Organic acids and flavins were quantified by high-performance liquid chromatography. Organic acids were separated on an Ultra AQ C18 column (Restek, USA) using an eluent consisting of 1% acetonitrile and 99% 50 mM aqueous K_3_PO_4_ (pH 2.5) and detected at 210 nm. Flavins were separated on an Acclaim 120 C18 column (Thermo Fisher Scientific, USA) with an eluent consisting of 15% ethanol and 85% 50 mM aqueous ammonium acetate (pH 6.0) and detected by fluorescence (excitation, 462 nm; emission, 525 nm).

Fe(II) production from Fe(III) reduction was monitored as previously described (21, 33). Briefly, culture aliquots were extracted in 0.5 N HCl and Fe(II) concentrations were determined using the ferrozine assay (30). Cell numbers in Fe(III) oxide-grown cultures were determined using a modified acridine orange direct count method previously described (42). Culture samples (1 mL) were fixed with glutaraldehyde (2.5% final concentration). Fixed samples were mixed with 5.8 mL of sterile oxalate solution (28 g ammonium oxalate and 15 g oxalic acid per liter) and 0.1 mL FeCl_2_ (0.26 M) to dissolve Fe(III) oxide and associated iron precipitates. A 2-mL subsample was then stained with acridine orange (0.118%) for 2 min and vacuum-filtered through a black membrane filter (0.2 μm pore size). Filters were examined using a Zeiss confocal scanning laser microscopy (Zeiss LSM 900, Zeiss, Germany) at a wavelength of 490 nm.

## Data Availability

Raw sequence data have been deposited in the National Center for Biotechnology Information (NCBI) Sequence Read Archive (SRA) under BioProject accession PRJNA1477714 and SRA accession numbers SRR39122162–SRR39122167. Other data that support the findings of this study are available from the corresponding author upon reasonable request.

## SUPPLEMENTAL MATERIAL

Fig. S1 and S2 mbio.02206-26-s0001.docx Lack of growth on trace sulfate and alignment of flavin transport genes.

Table S1 mbio.02206-26-s0002.xlsx Differential gene expression for growth on sulfate or Fe(III).

ASM does not own the copyrights to Supplemental Material that may be linked to, or accessed through, an article. The authors have granted ASM a non-exclusive, world-wide license to publish the Supplemental Material files. Please contact the corresponding author directly for reuse.
