# Supplementary material for: Fe(III) oxide respiration by a Desulfovibrio species lacking canonical outer-membrane electron transfer systems
Source: mBio. 2026 Oct 9;17(11):e02206-26. doi: 10.1128/mbio.02206-26 (PMC13753845; doi:10.1128/mbio.02206-26)
Supplement: Supplementary file 1 [file mbio.02206-26-s0001.docx]

**Supplemental Material**

**Fe(III) Oxide Respiration by a *Desulfovibrio* species Lacking Canonical Outer-Membrane Electron Transfer Systems**

Qin Cheng ^a,b,c^, Dawn E. Holmes ^d,e^, Dake Xu ^a,b,*^, Derek R. Lovley ^a,d,*^

a. Electrobiomaterials Institute, Key Laboratory for Anisotropy and Texture of Materials (Ministry of Education), Northeastern University, Shenyang, China
b. State Key Laboratory of Digital Steel, Northeastern University, Shenyang, 110819, P. R. China
c. College of Medicine and Biological Information Engineering, Northeastern University, Shenyang, China
d. Department of Microbiology, University of Massachusetts, Amherst, MA, USA
e. Department of Physical and Biological Science, Western New England University, Springfield, Massachusetts. USA

*Correspondence: [xudake@mail.neu.edu.cn](mailto:xudake@mail.neu.edu.cn) (D. Xu), [dlovley@umass.edu](mailto:dlovley@umass.edu) (D.R. Lovley)

**FIG S1** Lack of strain JY growth supported by trace sulfate reduction in NB medium with lactate as the electron donor. (A) Metabolism of strain JY in medium without Fe(III) oxide. (B) Cell counts over time for the incubation shown in panel A. Representative fluorescence microscopy images of cells collected on days 0, 3, and 6 are shown above the corresponding data points. (C) Metabolism after the incubation in panel A was used as a 10% inoculum into fresh medium without Fe(III) oxide. (D) Cell counts over time for the incubation shown in panel C. Representative fluorescence microscopy images of cells collected on days 0, 3, and 6 are shown above the corresponding data points.

**FIG S2** Multiple sequence alignment of the proteins encoded by strain JY proteins genes K9F62_11825 and K9F62_04930 with the flavin exporter homolog Bfe from *Shewanella oneidensis*. Residues highlighted in red are completely conserved, and residues boxed in blue are 80% conserved. The alignment was generated using ClustalW and visualized with ESPript.
